# Mindfulness-based cognitive therapy for children (MBCT-C) on internalizing, externalizing, and attention problems in children from single-parent families: A study protocol for a crossover randomized clinical trial

**DOI:** 10.1371/journal.pone.0354802

**Published:** 2026-08-06

**Authors:** Sara Jamali, Isaac Rahimian-Boogar, Mojtaba Habibi Asgarabad

**Affiliations:** 1 Department of Clinical Psychology, Faculty of Psychology and Educational Sciences, Semnan University, Semnan, Iran; 2 Department of Psychology, Norwegian University of Science and Technology, Trondheim, Norway; 3 Department of Psychology, Faculty of Humanities and Social Sciences, Istinye University, Istanbul, Turkey; Hochschule Niederrhein - Campus Mönchengladbach: Hochschule Niederrhein - Campus Monchengladbach, GERMANY

## Abstract

**Objective:**

Mindfulness-based cognitive therapy for children (MBCT-C) is an evidence-based intervention that requires further investigation in relation to its effectivness across diverse populations and prolonged follow-up durations. The present study aimed to develop a study protocol to evaluate MBCT-C in addressing internalizing and externalizing problems, as well as attention problems, among children from single-parent families.

**Method:**

A two-arm randomized controlled trial (RCT) using crossover conditions is proposed. The intervention condition will receive twelve weekly sessions of MBCT-C group therapy, while the waitlist (WL) condition will initially serve as a control and then cross over to receive MBCT-C. A total of seventy-six children (aged 10–12) will be randomly allocated to the two study arms. Assessments will be conducted at baseline, post-intervention, and at 3-, 6-, 9-, and 12-month follow-ups. Participants will be assessed by the Kiddie Schedule for Affective Disorders and Schizophrenia-Present and Lifetime Version (K-SADS-PL), and will respond to the Child Behavior Checklist (CBCL), Pediatric Symptom Checklist (PSC), Clinical Global Impressions (CGI), Mindfulness Inventory for Children and Adolescents (MICA), MBCT-C Adherence Scale (MBCT-C-AS), Mindfulness-Based Cognitive Therapy for Child and Parent Evaluation Questionnaire (MBCT-C–CPEQ). Data will be analyzed using a mixed regression model in STATA 18.

**Results:**

As this is a study protocol, no results are available to report at this time.

**Conclusions:**

This study protocol is the first attempt to evaluate the long-term effectiveness of MBCT-C using a crossover RCT that will help develop interventions tailored to internalizing, externalizing, and attention problems among children from single-parent families.

## Introduction

More than two billion children currently live worldwide [1], a number projected to increase by over 60 million in the next two decades [2]. Parent–child interactions [3], parenting practices [4,5], and family structure [6] are recognized as key influences on children’s development and well-being [7]. Political changes, socio-cultural issues, and the loss of one parent have been associated with an increase in single-parent families, which currently account for approximately 6.8% of families worldwide [8]. According to the Statistical Center of Iran, 7.2% of households, or more than one million, 753,960 households, are single-parent households, of which 17% have a male head and 83% have a female head [9]. Evidence shows that parental absence and conflict between separated or divorced parents negatively impact children’s mental and physical health, development, and peer relationships throughout their lives [10].

Disrupted family relationships and the absence of a parent are associated with developmental and mental health challenges in children [11]. One such at-risk group is children from single-parent families, who are at risk of neglect, emotional abuse, and physical abuse due to limited resources and reduced parental capacity [12]. Attention problems and the lack of emotion regulation are also linked to chronic stress in such children. Studies have demonstrated that both internalizing (anxiety and depression) and externalizing (aggression and conduct) problems are prevalent in this population [13].

Considerable evidence supports the effectiveness of cognitive-behavioral interventions in adults [14,15]. However, there are significant challenges associated with applying these interventions to children [16]. In particular, given that these interventions are developed based on metacognition, verbal reasoning, causal and logical thinking, and understanding of emotions, children often struggle to grasp their core concepts [17].

Mindfulness-based cognitive therapy (MBCT) is one of the most promising methods of addressing psychopathology [18]. Earlier studies revealed that mindfulness-based interventions can be used as appropriate psychological interventions to treat mental health problems in children and adolescents [19]. he aim of mindfulness-based interventions is to increase awareness of the present moment and to focus attention on thoughts, emotions, and physical sensations, helping them cope with negative experiences [20]. Thus, these interventions have not only broadened traditional treatments, but have also shown new solutions to address mental disorders.

MBCT for Children [MBCT-C; 21] was developed as a child-specific form of MBCT to provide tailored therapy for children. The protocol was tested across different populations to determine its effectiveness for behavioral issues [18,22–24]. Esmailian, Tahmasian [25] revealed that the MBCT-C have a considerable effectivness on depression, anxiety levels and anger problems among children of divorced parent during 2-month follow-up. Additionally, the increased emotional resilience and mindfulness following this intervention suggest that MBCT-C is a useful intervention for enhancing emotional well-being in children with divorced parents. Findings from another study examining children with cancer found that MBCT-C was highly effective in minimizing internalizing symptoms and attention problems [26]. A study by Wright, Roberts [18] examined the effectiveness of MBCT-C as a preventive intervention for internalizing behavioral problems and found preliminary evidence to support its effectiveness. Findings also suggested that MBCT-C may be a practical and clinically applicable school-based prevention program for reducing internalizing symptoms. Another study of the effectiveness of MBCT on generalized anxiety disorder in elementary school girls found that mindfulness training reduced worry and anxiety [27]. Finally, there is evidence support that MBCT-C can be effective in addressing attention and behavioral disorders as well as anxiety in children [28].

The developmental factors are central to the adaptation of mindfulness-based interventions in children. Semple and Lee [21] that the kind of learning activities that grow out of the tangible sensory experiences allows mindful awareness by enabling children to interact more successfully both with their interior world as well as with the world immediately around them. Since children generally have less developed attentional and memory abilities in comparison to adults, the more developmentally suitable intervention sessions seem to be shorter and repetitive [29]. Furthermore, an adult psychotherapy frequently is based on an abstract thought and verbal emotional understanding, but the emotional experience of children is more shaped in the context of their immediate family and the school. In line with this, parents participation in introductory and review boards, alongside organized communication about home practice has been pointed as an essential element in improving the practice and treatment results in kid-centered mindfulness programs [21].

Based on these developmental concepts, mindfulness-based cognitive therapy with children (MBCT-C) has been precisely developed as a group-based approach to youth between the ages of 9 and 13 years [28], with a view of enhancing social-emotional resilience via development of mindful awareness (Semple et al., 2010). Empirical evidence indicates that there is a significant relationship between attentional problems and behavioral issues in children with high levels of anxiety showing improvements in anxiety and behavioral symptoms after attending MBCT-C [28]. Further research has also supported the effectiness of MBCT-C with anxious child samples. As an example, Shetty, Kongasseri [30] revealed that MBCT-C had been linked to more anxiety and emotion suppression improvements, and emotion regulation. There is also neurobiological evidence that MBCT-C can be associated with more brain activity in interoceptive awareness and internal stimuli processing in anxious youth with a family history of bipolar disorder [31].

Although such encouraging results were obtained, the present evidence base is still remains limited and heterogeneous. Pilot research showed that mindfulness techniques can be effectively implemented in children and potentially reduce symptoms of anxiety, especially the symptoms of attentional functioning at the baseline, however the same studies also revealed the relative lack of rigorous research in pediatric cohorts in comparison to adults [32]. Syeda and Andrews [24] identified significant improvements reported by parents in anxiety symptoms in children receiving MBCT-C, and these improvements were maintained at one-month follow-up, but their research limited generalizability due to the small sample size. Conversely, only significant effects of MBCT-C and cognitive behavioral therapy were found by Wright, Roberts [18] on anxiety, depression and quality of life, but there were no significant differences between mindfulness and sustained attention. These discrepancies provoke significant questions about how exactly MBCT-C has its action and why larger, methodologically sound studies are necessary to make the processes of attention and mindfulness the sources of therapeutic change.

Although the current evidence on the topic of MBCT-C is promising, the available research is still limited in terms of small sample sizes, a comparatively short follow-up, and a lack of randomized controlled trials. Moreover, there is a paucity of research that has specifically investigated the efficacy of MBCT-C in susceptible groups of individuals, including children in single parent families, although there is extensive evidence to suggest that children in single parent families are at a high risk of developing emotional, behavioral, and attentional challenges.

To close these gaps, the current study will involve a Crossover randomized controlled trial (RCT) with a 12-month follow-up to determine the impact of MBCT-C in children from single-parent families. A 12-month follow-up period was selected to allow for the evaluation of both immediate and longer-term effects of MBCT-C across internalizing, externalizing, and attention outcomes. Previous research on mindfulness-based interventions in children and adolescents suggests that extended follow-up periods are necessary to assess the durability of treatment effects and the emergence of delayed benefits [28]. A shorter follow-up duration may not adequately capture the maintenance or decline of treatment gains, whereas longer follow-up periods may increase the risk of attrition and introduce confounding influences related to developmental changes. Therefore, a 12-month timeframe represents a balanced approach to evaluating sustained intervention effects while maintaining methodological feasibility. The hypothesis is that children who receive MBCT-C will show substantial positive changes in central mindfulness dimensions, including self-acceptance, present-moment awareness, and metacognitive awareness. Moreover, participation in MBCT-C is expected to be associated with reductions in internalizing and externalizing problems. Lastly, the intervention is expected to produce meaningful improvements in attentional functioning. Potential gender differences will also be explored to determine whether treatment effects vary between boys and girls.

## Method

### Design

**Fig 1:**
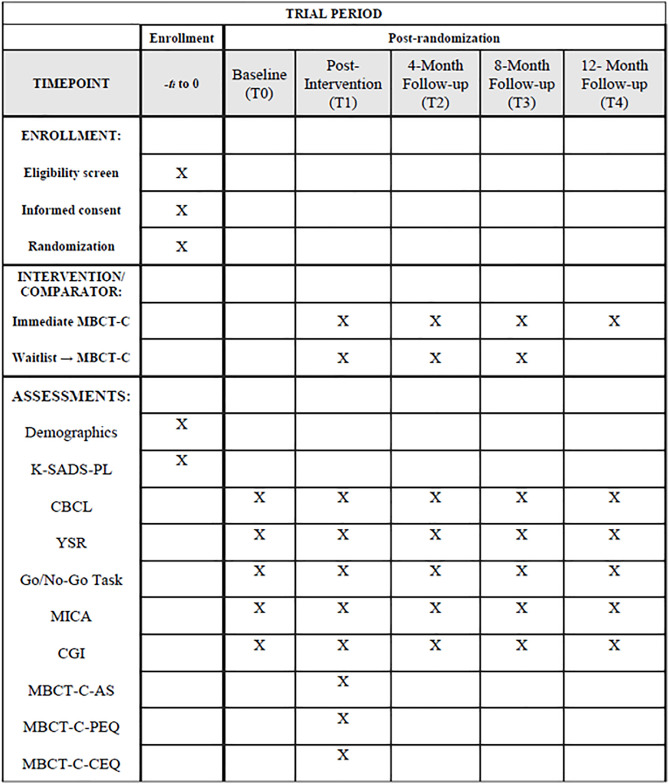
Participant timeline: Schedule of enrollment, interventions, and assessments. Note: SPIRIT schedule of enrollment, interventions, and assessments. Abbreviations: K-SADS-PL, Kiddie Schedule for Affective Disorders and Schizophrenia-Present and Lifetime Version; CBCL, Child Behavior Checklist; YSR, Youth Self-Report; CGI, Clinical Global Impressions; MICA, Mindfulness Inventory for Children and Adolescents; MBCT-C-AS, Mindfulness-Based Cognitive Therapy for Children Adherence Scale; MBCT-C-PEQ, Parent Evaluation Questionnaire; MBCT-C-CEQ, Child Evaluation Questionnaire.

**Fig 2:**
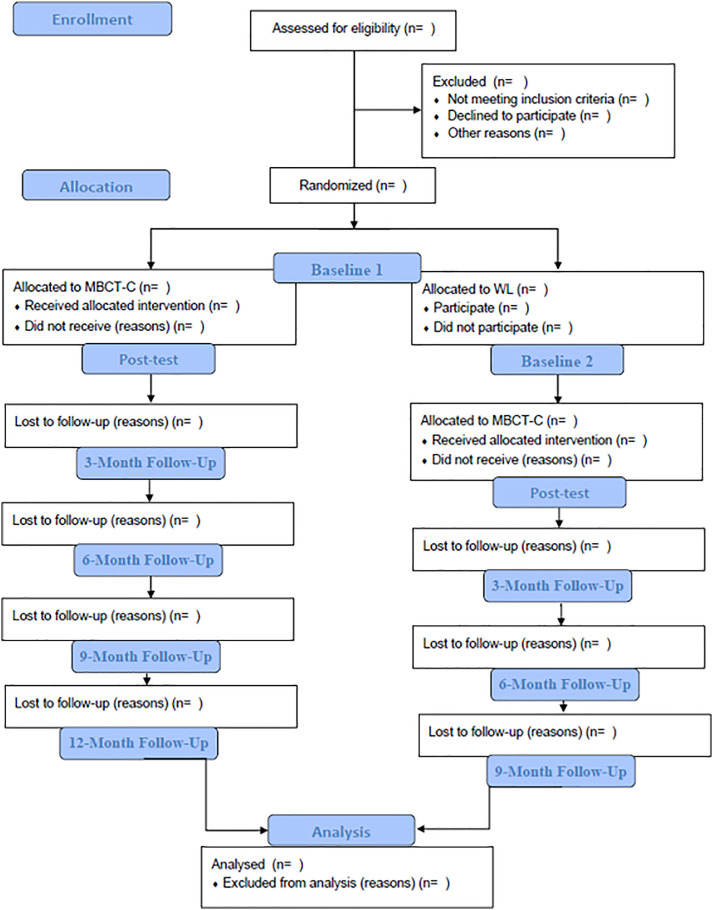
Consort diagram of this proposed study protocol.

A randomized controlled trial (RCT) with crossover design will be used in this study. The study will involve random assignment of the participants into the mindfulness-based cognitive therapy of children (MBCT-C) intervention group and waitlist control group (WLCG). The participants assigned to the MBCT-C group will undergo a baseline assessment (T1) and will then be subjected to the 12 weekly sessions of the MBCT-C. The outcome measures will be done at the end of the intervention (T2), and after 3-, 6-, 9-, and 12-months (T3-T6). Respondents who are to be part of the WLCG will undergo a baseline assessment at the time of entering the study (T1) and will be kept on the waitlist at the start of the trial. After the MBCT-C group has gone through the intervention period, the participants of WLCG will be given the same 12-session MBCT-C program. The WLCG will undergo post-intervention and follow-up assessment at T3 and 3-, 6-, and 9-month follow-ups respectively (T4-T6). Figs 1 and 2 show the general study design and the evaluation schedule.

### Participants

The study will include 76 children aged 10–12 years from single-parent families (either the mother or the father), encompassing both genders in Semnan province, Iran. To be eligible for inclusion, participants must demonstrate borderline or clinical levels of attention problems (T-score ≥ 57) and internalizing difficulties (T-score ≥ 60) on a minimum of two out of four subscales from either the Child Behavior Checklist (CBCL) or Youth Self-Report (YSR) [26]. Exclusion criteria include: (a) significant cognitive impairment or developmental disorder, (b) terminal illness with a prognosis of less than six months, (c) chronic psychiatric disorders (e.g., schizophrenia or bipolar spectrum) or neurodevelopmental disorders per DSM-5, assessed via the Kiddie Schedule for Affective Disorders and Schizophrenia-Present and Lifetime Version [K-SADS-PL; 33], (d) change in parental status to an orphan or a two-parent household, and (e) chronic medical conditions impacting psychosocial functioning. A clinical psychologist will conduct psychiatric evaluations using the K-SADS-PL. Written informed consent will be obtained from parents, and children will provide informed assent.

### Sample size

The sample size for this study will be determined using G*Power software. According to an a priori power analysis, it is assumed that the smallest clinically meaningful difference corresponds to a small effect size (*Cohen’s d* = .20 [34]), with effect sizes smaller than this considered clinically insignificant. Taking into account the three assessment time points (pre-intervention, post-intervention, and follow-up) and the number of dependent variables, a sample size of 38 participants per condition is necessary (*alpha* = .05, *effect size* = .20, *power* = .95). To account for a 20% dropout rate, the total sample size will be 76 children, who will be randomly assigned to either the MBCT-C group (n = 38) or the WLCG group (n = 38).

### Procedure

Registration for this study was completed through the Iranian Registry of Clinical Trials (IRCT20240729062574N1). Recruitment will commence in February 2026 and continue until 76 children are enrolled. Participants will be matched by age and gender and randomly assigned to either the MBCT-C or WLCG group using a computer-generated randomization sequence. The intervention will be conducted in small groups of 8–10 children to maximize group dynamics, and all of the sessions will be conducted by the first author. The data will be collected at the baseline (T1), post-intervention (T2 in the case of MBCT-C, T3 in the case of WLCG), and follow-up (T3-T6 in the case of MBCT-C, T4-T6 in the case of WLCG). Self-report measures (e.g., CBCL, YSR) will be given to the participants by research associates and will be facilitated to make sure that the research participants complete them correctly. At enrollment, semi-structured interviews with the use of the K-SADS-PL will be carried out to verify eligibility. The research design is a single-blind design, whereby the participants do not know certain information about the other group in order to reduce bias. The data will be analyzed by an independent statistician who will be blinded to the group allocations. All the sessions will be audio-taped to make sure that the intervention fidelity is ensured, and recordings will be checked by a clinical psychologist to give feedback to the facilitator. Weekly email notifications will be used to motivate participants to provide data and fill surveys.

### Feasibility and acceptability measures

In order to determine the efficacy of MBCT-C in alleviating internalizing and externalizing problems and enhancing attention in children in single-parent families, a number of important indices will be derived. The first one will be the attrition rate (i.e., the percentage of children who stop receiving treatment) to evaluate the viability of the MBCT-C. The second one will be the engagement and response rates. Moreover, two indices will be tested to determine the acceptability of MBCT-C: first, the children will be evaluated according to their satisfaction with the treatment by MBCT-C-CEQ and then the parent satisfaction will be assessed by MBCT-C-PEQ.

### Interventions

MBCT-C is a manualized group intervention that occurs over 12 weeks. MBCT-C, adapted to children aged 10–12 years, includes mindfulness meditation base with cognitive-behavioral intervention and assists children in managing emotional (e.g., relational and/or familial conflict) and behavioral (e.g., attention problems) difficulties 39 The program aims at fostering awareness of feelings; an intentional focus of attention and emotion regulation through structured activities that are tailored according to the developmental needs of a young child. The same MBCT-C protocol is followed for each session: mindfulness meditation practices, guided sensorial exercises and psychoeducational components. Some of these are: (a) mindfulness of breath practices for enhancing attention and present-moment awareness, (b) body-scan exercises to help focus on physical sensations, (c) sensory-focused activities (e.g., mindful eating, or mindful listening) to help focus on intrapsychic events including thoughts and feelings without judgment; and (d) guided inquiry into intrapsychic events such as thoughts and feelings in an open-minded way [21]. The activities and games will be tailored to the level of the children, and allowing them to learn how to do so through hands-on, playful activities. So children can learn practices of mindful movement or storytelling to connect the concepts of mindfulness with their day-to-day activities.

### Measures

***The Kiddie Schedule for Affective Disorders and Schizophrenia-Present and Lifetime Version (K-SADS-PL)*** is a semi-structured clinical interview to determine 32 psychiatric diagnoses in children and adolescents and includes both parents and children [33]. Diagnostic outcomes derived from this instrument are treated as categorical variables in the analyses. The K-SADS-PL has shown high reliability and validity in the diagnosis of mental health disorders among the youth, and its concurrent validity of current disorders is high. Besides, the test-retest reliability of most diagnoses is good to excellent [35]. The K-SADS-PL Persian version has been proven to be valid and reliable in the Iranian setting. The validation study carried out by Shahrivar, Kousha [36], in Iran revealed that the specificities were over 81% on all disorders, and the sensitivities were between 75 and 100% on the majority of the major diagnoses. Kappa agreement coefficients of most diagnoses were above.4 which illustrates acceptable to strong concordance with test-retest reliability coefficients ranging between.38 and.87.

***The Child Behavior Checklist (CBCL/6–18) and the Youth Self-Report (YSR/11–18)*** are the two tools that assess behavioral and emotional issues in children and adolescents [37]. The CBCL is filled in by the parents or caregivers and the YSR is filled in by the youth. The CBCL has 113 items that are disseminated into eight syndrome scales, which include withdrawal/depression, aggressive behavior, depression/anxiety, somatic complaints, rule-breaking behavior, thought problems, social problems, and attention issues. These scales are divided into two broad categories; internalizing and externalizing behaviors. A study by Abedini, Habibi [26] has revealed that the Persian version of the CBCL/6–18 is quite reliable, with Cronbach alpha coefficients of between.72 and.89. Furthermore, YSR as a youth assessment scale that contains 112 questions aimed at testing youth between the age of 11 and 18; (Fadaei et al., 2009), a validated Persian version of the YSR was employed in the research.

***The Pediatric Symptom Checklist (PSC)*** is a brief tool to determine emotional and behavioral difficulties in children [38]. This checklist measures a wide range of psychiatric symptoms in children and emphasizes the importance of early intervention and appropriate support. The original version of PSC contains 35 items that ask about the child’s internalization, externalization, and attention problems over the past few months. Items are rated as “*Never*,” “*Sometimes*,” or “*Often*” (scored as 0, 1, or 2). The total score ranges between 0 and 75. These scores are treated as continuous variables in the statistical analyses. Lower scores indicate lower levels of difficulty. Scores derived from the PSC are treated as continuous variables in the statistical analyses. The internal consistency of the PSC was excellent, with a *Cronbach’s alpha* of.89 [39].

***The Clinical Global Impressions (CGI)*** have been developed for clinicians to assess illness severity, treatment outcomes, and the effectiveness of interventions in psychiatry [40]. This instrument consists of three main items, each with a 2-item rating scale, that evaluate disease severity, improvement, or overall change in a simple and efficient manner. The scoring of this instrument is based on a 7-point Likert scale. The CGI-Severity and CGI-Improvement scales are continuous measures and are treated as continuous variables in the analyses. Therapists were provided with written protocols for the CGI as part of their project training, and the inter-rater reliability for the CGI was found to be high (*weighted kappa* = .52) [41].

***The Mindfulness Inventory for Children and Adolescents (MICA)*** is a 25-item self-report tool for assessing mindfulness in children and adolescents aged 8–18 [42]. Specifically, the MICA is designed to be (a) theory-driven, thereby enhancing its construct validity; (b) positively worded, thereby strengthening both construct and content validity; (c) comparatively concise; (d) readable and pertinent to children as young as eight years old; and (e) multifactorial, permitting the evaluation of multiple facets of mindfulness [43]. Items are rated on a structured scale and summarized into total scores. These scores are treated as continuous variables. An unpublished short version of MICA, developed by Semple and Goodman, will be used in this study.

The internal consistency coefficients of this short version varied according to age, with alpha for children ranging from.56 to.81, with a mean alpha of.69, and adolescent alphas ranging from.64 to.83 with a mean alpha of.74. Considering that this instrument has been recently designed and has not yet been officially published, before starting this study, this scale will be translated and after cultural adaptation, the psychometric characteristics of its Persian version will be reported.

***The MBCT-C Adherence Scale (MBCT-C-AS)*** is an unpublished clinician-rated adherence measure developed by Semple and Sears. MBCT-C-AS comprising 20 items, each rated using a three-point Likert-type scale (0 = *not at all*, 1 = *slight-inconsistent*, 2 = *clear and consistent*), the scale evaluates therapist performance in areas such as fostering group cohesion, facilitating non-directive participation, providing developmentally appropriate education about anxiety or depression, explaining the rationale for MBCT-C, using metaphors, implementing breathing exercises, and conducting cognitive, movement-based, and sensory awareness activities. We will use the MBCT-C-AS to informally monitor the treatment’s fidelity. Scores derived from the MBCT-C-AS are treated as continuous variables.

***Mindfulness-Based Cognitive Therapy for Parent Evaluation Questionnaire (MBCT-C–PEQ)*** designed by Semple & Lee (2007), is a 15-item instrument designed to evaluate parental satisfaction with the MBCT-C program. The scale ranges from 1 (*strongly disagree or very unhelpful*) to 5 (*strongly agree or very helpful*). Total scores can range from a minimum of 15 to a maximum of 75, with elevated scores indicate increased satisfaction levels. These scores are analyzed as continuous variables. Although no standardized norms exist, total scores may be interpreted using suggested cut-offs: 65–75 = *excellent*, 50–64 = *good*, 35–49 = *fair*, and 15–34 = *poor* satisfaction. For larger samples (n ≥ 40), item-level means may also be reported; otherwise, total scores or response categories can be used for group-level interpretation [21].

***Mindfulness-Based Cognitive Therapy for Children – Child Evaluation Questionnaire (MBCT-C–CEQ)*** is a 10-item measure assessing satisfaction and engagement with the MBCT-C program ([21]. A 5-point rating scale is used, ranging from 1 (*strongly disagree or very unhelpful*) to 5 (*strongly agree or very helpful*). The overall score is between 10 and 50 where the higher the score the more the satisfaction, categories: 42–50 (*excellent*), 35–41 (*good*), 25–34 (*fair*), 10–24 (*poor*). These scores are treated as continuous variables for analysis. The MBCT-C-CEQ consists of eight open-ended questions, the answers are coded in themes like perceived benefits and suggestions that are summarized by frequencies/frequency of key points in quotes [21].

### Data analyses

The entire analysis will be conducted on the STATA version 18 software. To begin with, descriptive statistics, box plots and scatter plots will be used to analyze the accuracy and distribution patterns of the data sets. 5 percent trimmed mean will be used to compare means to determine outliers. In case of no significant issues, all cases will be stored and regular cleaning will be used.

All analyses will be done using an intention-to-treat approach. Linear mixed-effects models will be used to analyze the changes at baseline, postintervention, and 3-, 6-, 9-, and 12-month follow-ups. All assumptions for the linear mixed-effects models will be assessed before interpreting the results. Normality of residuals will be checked with Q-Q plots and the Shapiro-Wilk test, while homoscedasticity will be evaluated using residuals versus fitted value plots. Multicollinearity among predictors will be examined with variance inflation factors. The independence assumption will be addressed by modeling repeated measurements within participants through random effects structures. If any assumptions are violated, alternative approaches such as generalized linear mixed models, robust standard errors, data transformations, or non-parametric analyses will be considered, based on the nature of the violation.

The models presuppose a crossover design where the waitlist group is provided with MBCT-C at the end of the waiting period. The primary variables will be treatment condition, time, and period, and their interaction. Covariates will include age, gender, baseline scores, and parental stress. The models will adjust for carryover effects, repeated measures, clustering within groups and sequence effects. Model fit will be evaluated via residual diagnostics, which include inspecting residual plots, assessing normality and homoscedasticity, and comparing different covariance structures using Akaike’s Information Criterion and Bayesian Information Criterion. The findings will indicate adjusted mean changes and 95% confidence intervals and effect sizes.

Sensitivity analyses will be done using generalized estimating equations to estimate population effects at various time points. The results of internalizing, externalizing, and attention will be analyzed using a multivariate mixed model and will explain the correlations between them. Several comparison problems will be solved either through Bonferroni correction or hierarchical testing.

Clinical significance will be determined through the reliable change index and responder analysis according to CBCL, PSC and CGI thresholds. The proportion of the participants who will have significantly improved will be compared in both crossover sequences at the end of the intervention and during the follow-up. Maximum likelihood estimation will be used to deal with missing data, assuming that they are missing at random. The findings are in line with the CONSORT requirements of crossover trials.

### Ethical considerations

In line with the Declaration of Helsinki and international research on clinical interventions, the entire procedure will be design as to full fill ethic principles. Written informed consent will be obtained from the children’s parents or legal guardians by the research staff. Furthermore, children will sign their own assent to participate in this study. Details of participation Involvement in this study is voluntary. Individuals will be informed that they are free to leave the study without repercussions. Records will be kept securely in accordance with the General Data Protection Regulation (GDPR). The data collected in this study will be de-identified and privacy will be preserved according to the General Data Protection Regulation. The participants and their parents will also be informed of the procedures for protecting data. Children and their parents will be advised of the aims and methods of the study prior to the commencement of data collection, and parents are free to raise any questions or concerns they might have.

### Current status

As of February 2026, participant recruitment for this crossover randomized clinical trial is actively ongoing and is anticipated to be completed by May 2026, with a target sample size of 76 children from single-parent families. Data collection commenced in December 2025 and is scheduled to continue until June 2026. Due to the crossover design, no participants will receive any study intervention until after recruitment is fully completed in May 2026. Consequently, outcome data related to the primary and secondary endpoints (internalizing, externalizing, and attention problems) remain uncollected at the time of protocol submission, and no study results have been generated or analyzed to date.

## Discussion

The suggested crossover RCT design will involve the effectiveness of MBCT-C in reducing internalizing, externalizing, and attention-related problems in children living in single-parent families. MBCT-C is a child-adapted form of MBCT, which is aimed at enhancing the present-moment awareness, emotional regulation skills, and cognitive control skills of children [21,44].

Prior research indicates that MBCT-C effectively mitigates anxiety, depression, and behavioral problems in adolescents [45,46]. MBCT-C enables children to observe their thoughts and feelings without judgment, and this can disrupt their maladaptive cognitive and emotional processes that contribute to the internalizing and externalizing symptoms [47]. Moreover, MBCT-C is expected to resolve the lack of attentional control, which is typical of children with emotional and behavioral problems [48].

The present study is specifically dedicated to children in single-parent families, the number of which is growing worldwide [49]. The findings may provide information on how mindfulness-based interventions can be adapted to better address the needs of children from single-parent families. Moreover, this study highlights the potentially broad impact of MBCT-C by targeting a range of outcomes, including internalizing symptoms, externalizing behaviors, and attention-related difficulties.

The study may also contribute to the existing literature [26,48,50] by examining potential mechanisms through which MBCT-C influences children’s psychological outcomes. For example, improvements in attentional regulation and emotional awareness may function as mediating processes and offer further insight into how this intervention exerts its effects [51].

This study could increase statistical power by employing within-group comparisons even with a relatively small sample size [52], a problem that is often controversial in parallel clinical trials. However, maintaining long-term participant engagement and eliminating potential carryover effects may be difficult. The current protocol will address these issues by incorporating waiting periods and randomized treatment orders.

Previous research suggests that MBCT-C may be effective in reducing depression and anxiety and improving emotion regulation across different populations [18,26,48]. Although direct evidence for the effects of MBCT-C on externalizing problems remains limited, mindfulness practices may support behavioral regulation and attentional control through their influence on executive functioning [53].

### Limitations

This proposed RCT is a strong framework for assessing the effectiveness of MBCT-C, but it is limited in several ways. To begin with, data accuracy can be biased by subjective measures, such as parent- and child-reported outcomes, which may be affected by recall errors or social desirability [54]. Objective data, such as physiological markers or directly observable behavior, would help increase reliability. Second, a crossover design is more statistically powerful, but such problems as participant dropout and non-adherence to the variables can bias results [55]. This should be addressed in future studies through fidelity checks and incentives. Third, the research findings may be limited in generalizability because the sample consisted of children from single-parent households with heterogeneous demographic characteristics [56].

## Conclusions

The findings of this study, by characterizing the effectiveness of MBCT-C, enable psychotherapists to implement this evidence-based intervention to treat internalizing problems, externalizing problems, and improve attention in single-parent children. In addition, the results of this study provide more real-time treatment for these children. Also, MBCT-C will reduce the likelihood of symptom recurrence in these children.

## Supporting information

S1 File English protocol. (PDF)

S2 File Persian protocol. (PDF)

S3 File SPIRIT checklist. (DOC)

S4 File Inclusivity in global research questionnaire. (DOCX)

## References

[1] World Population Review. Children in the World by Country. 2025 [cited 2025 Oct 11]. Available from: https://worldpopulationreview.com/country-rankings/children-in-the-world-by-country

[2] Department of Economic. World Population Prospects 2024: Summary of Results. Stylus Publishing, LLC; 2024.

[3] Kim JH, Schulz W, Zimmermann T, Hahlweg K. Parent–child interactions and child outcomes: evidence from randomized intervention. Labour Econ. 2018;54:152–71. doi: 10.1016/j.labeco.2018.08.003

[4] Kwon S, Shin ED, Bartell TR, Capan S. Parenting practices and well-being and health behaviors among young Asian American Children. JAMA Network Open. 2025;8(1):e2454516-e. doi: 10.1001/jamanetworkopen.2024.54516 10.1001/jamanetworkopen.2024.54516 PMC11731191 39804643

[5] Edrissi F, Havighurst SS, Aghebati A, Habibi M, Arani AM. A pilot study of the tuning in to kids parenting program in Iran for reducing preschool children’s anxiety. J Child Fam Stud. 2019;28(6):1695–702. doi: 10.1007/s10826-019-01400-0

[6] Lu Y, Zhang R, Du H. Family structure, family instability, and child psychological well-being in the context of migration: evidence from sequence analysis in China. Child Dev. 2021;92(4):e416–38. doi: 10.1111/cdev.13496 33410505

[7] Ani NC. The impact of family dynamics on children’s mental health: systematic review. Niger J Arts Hum. 2024;4(2).

[8] Chavda K, Nisarga V. Single parenting: impact on child’s development. J Indian Assoc Child Adolescent Mental Health. 2023;19(1):14–20. doi: 10.1177/09731342231179017

[9] Seraj Shirvan F, Latifnejad Ruodsari R, Tehrani H, Ebrahimipour H, Moradi M. Iranian single-child couples’ perceptions and experiences regarding childbearing incentives. Reprod Health. 2024;21(1):148. doi: 10.1186/s12978-024-01885-z 39420371 PMC11487753

[10] Amato PR, Patterson S, Beattie B. Single-parent households and children’s educational achievement: a state-level analysis. Soc Sci Res. 2015;53:191–202. doi: 10.1016/j.ssresearch.2015.05.012 26188447 PMC4508674

[11] Lut I, Woodman J, Armitage A, Ingram E, Harron K, Hardelid P. Health outcomes, healthcare use and development in children born into or growing up in single-parent households: a systematic review study protocol. BMJ Open. 2021;11(2):e043361. doi: 10.1136/bmjopen-2020-043361 33574152 PMC7880085

[12] Guo T, Jiang D, Kuang J, Hou M, Gao Y, Herold F, et al. Mindfulness group intervention improved self-compassion and resilience of children from single-parent families in Tibetan areas. Complement Ther Clin Pract. 2023;51:101743. doi: 10.1016/j.ctcp.2023.101743 36913906

[13] Yang Y, Shields GS, Zhang Y, Wu H, Chen H, Romer AL. Child executive function and future externalizing and internalizing problems: a meta-analysis of prospective longitudinal studies. Clin Psychol Rev. 2022;97:102194. doi: 10.1016/j.cpr.2022.102194 35964337

[14] Cuijpers P, Harrer M, Miguel C, Ciharova M, Papola D, Basic D, et al. Cognitive behavior therapy for mental disorders in adults: a unified series of meta-analyses. JAMA Psychiatry. 2025;82(6):563–71. doi: 10.1001/jamapsychiatry.2025.0482 40238104 PMC12004241

[15] Ahmadi R, Ahmadizadeh R, Hasani M, Saed O. Transdiagnostic versus construct-specific cognitive behavioural therapy for emotional disorders in patients with high anxiety sensitivity: a double-blind randomised clinical trial. Behav Change. 2021;38(3):177–92. doi: 10.1017/bec.2021.6

[16] Halder S, Mahato AK. Cognitive behavior therapy for children and adolescents: challenges and gaps in practice. Indian J Psychol Med. 2019;41(3):279–83. doi: 10.4103/IJPSYM.IJPSYM_470_18 31142932 PMC6532387

[17] Rapley HA, Loades ME. A systematic review exploring therapist competence, adherence, and therapy outcomes in individual CBT for children and young people. Psychother Res. 2019;29(8):1010–9. doi: 10.1080/10503307.2018.1464681 29683046 PMC6763333

[18] Wright KM, Roberts R, Proeve MJ. Mindfulness-Based Cognitive Therapy for Children (MBCT-C) for prevention of internalizing difficulties: a small randomized controlled trial with Australian primary school children. Mindfulness. 2019;10(11):2277–93. doi: 10.1007/s12671-019-01193-9

[19] Featherston R, Barlow J, Song Y, Haysom Z, Loy B, Tufford L, et al. Mindfulness-enhanced parenting programmes for improving the psychosocial outcomes of children (0 to 18 years) and their parents. Cochrane Database Syst Rev. 2024;1(1):CD012445. doi: 10.1002/14651858.CD012445.pub2 38197473 PMC10777456

[20] Zhang D, Lee EKP, Mak ECW, Ho CY, Wong SYS. Mindfulness-based interventions: an overall review. Br Med Bull. 2021;138(1):41–57. doi: 10.1093/bmb/ldab005 33884400 PMC8083197

[21] Semple RJ, Lee J. Mindfulness-based cognitive therapy for anxious children: A manual for treating childhood anxiety: New Harbinger Publications; 2007.

[22] LaGue A, Eakin G, Dykeman C. The impact of mindfulness-based cognitive therapy on math anxiety in adolescents. Prev School Failure: Alternative Educ Child Youth. 2019;63(2):142–8. doi: 10.1080/1045988x.2018.1528966

[23] Lee J, Semple RJ, Rosa D, Miller L. Mindfulness-based cognitive therapy for children: results of a pilot study. J Cogn Psychother. 2008;22(1):15–28. doi: 10.1891/0889.8391.22.1.15

[24] Syeda MM, Andrews JJW. Mindfulness-based cognitive therapy as a targeted group intervention: examining children’s changes in anxiety symptoms and mindfulness. J Child Fam Stud. 2021;30(4):1002–15. doi: 10.1007/s10826-021-01924-4

[25] Esmailian N, Tahmasian K, Dehghani M, Mootabi F. Effectiveness of mindfulness-based cognitive therapy on depression symptoms in children with divorced parents. J Clin Psychol. 2013;5(3):47–57. doi: 10.22055/jacp.2015.12003

[26] Abedini S, Habibi M, Abedini N, Achenbach TM, Semple RJ. A randomized clinical trial of a modified mindfulness-based cognitive therapy for children hospitalized with cancer. Mindfulness. 2020;12(1):141–51. doi: 10.1007/s12671-020-01506-3

[27] Tang G, Chen B, Wu M, Sun L, Fan R, Hou R, et al. Effectiveness of mindfulness-based cognitive therapy for treating generalized anxiety disorder and the moderating influence of abuse during childhood: a randomized controlled trial. J Affect Disord. 2025;379:510–8. doi: 10.1016/j.jad.2025.02.103 40037493

[28] Semple RJ, Lee J, Rosa D, Miller LF. A randomized trial of mindfulness-based cognitive therapy for children: promoting mindful attention to enhance social-emotional resiliency in children. J Child Fam Stud. 2009;19(2):218–29. doi: 10.1007/s10826-009-9301-y

[29] Semple RJ. Does mindfulness meditation enhance attention? A randomized controlled trial. Mindfulness. 2010;1(2):121–30. doi: 10.1007/s12671-010-0017-2

[30] Shetty R, Kongasseri S, Rai S. Efficacy of mindfulness based cognitive therapy on children with anxiety. J Cogn Psychother. 2020;34(4):306–18. doi: 10.1891/JCPSY-D-20-00014 33372125

[31] Strawn JR, Cotton S, Luberto CM, Patino LR, Stahl LA, Weber WA, et al. Neural function before and after mindfulness-based cognitive therapy in anxious adolescents at risk for developing bipolar disorder. J Child Adolesc Psychopharmacol. 2016;26(4):372–9. doi: 10.1089/cap.2015.0054 26783833 PMC4876535

[32] Semple RJ, Reid EFG, Miller L. Treating anxiety with mindfulness: an open trial of mindfulness training for anxious children. J Cogn Psychother. 2005;19(4):379–92. doi: 10.1891/jcop.2005.19.4.379

[33] Kaufman J, Birmaher B, Brent D, Rao U, Flynn C, Moreci P, et al. Schedule for Affective Disorders and Schizophrenia for School-Age Children-Present and Lifetime Version (K-SADS-PL): initial reliability and validity data. J Am Acad Child Adolesc Psychiatry. 1997;36(7):980–8. doi: 10.1097/00004583-199707000-00021 9204677

[34] Cohen J. Statistical power analysis. Curr Directions Psychol Sci. 1992;1(3):98–101. doi: 10.1111/1467-8721.ep10768783

[35] Van Dam NT, van Vugt MK, Vago DR, Schmalzl L, Saron CD, Olendzki A, et al. Mind the hype: a critical evaluation and prescriptive agenda for research on mindfulness and meditation. Perspect Psychol Sci. 2018;13(1):36–61. doi: 10.1177/1745691617709589 29016274 PMC5758421

[36] Shahrivar Z, Kousha M, Moallemi S, Tehrani-Doost M, Alaghband-Rad J. The reliability and validity of kiddie-schedule for affective disorders and schizophrenia - present and life-time version - Persian Version. Child Adolesc Ment Health. 2010;15(2):97–102. doi: 10.1111/j.1475-3588.2008.00518.x 32847248

[37] Achenbach TM, Dumenci L, Rescorla LA. Ratings of relations between DSM-IV diagnostic categories and items of the CBCL/6-18, TRF, and YSR. Burlington, VT: University of Vermont; 2001. pp. 1–9.

[38] Jellinek MS, Murphy JM, Robinson J, Feins A, Lamb S, Fenton T. Pediatric symptom checklist: screening school-age children for psychosocial dysfunction. J Pediatrics. 1988;112(2):201–9. doi: 10.1016/S0022-3476(88)80056-8 10.1016/s0022-3476(88)80056-8 3339501

[39] Reijneveld SA, Vogels AGC, Hoekstra F, Crone MR. Use of the pediatric symptom checklist for the detection of psychosocial problems in preventive child healthcare. BMC Public Health. 2006;6:197. doi: 10.1186/1471-2458-6-197 16872535 PMC1550396

[40] Busner J, Targum SD. The clinical global impressions scale: applying a research tool in clinical practice. Psychiatry (Edgmont). 2007;4(7):28–37. 20526405 PMC2880930

[41] Toolan C, Holbrook A, Schlink A, Shire S, Brady N, Kasari C. Using the Clinical Global Impression scale to assess social communication change in minimally verbal children with autism spectrum disorder. Autism Res. 2022;15(2):284–95. doi: 10.1002/aur.2638 34800004 PMC8821201

[42] Brown KW, West AM, Loverich TM, Biegel GM. Assessing adolescent mindfulness: validation of an adapted Mindful Attention Awareness Scale in adolescent normative and psychiatric populations. Psychol Assess. 2011;23(4):1023–33. doi: 10.1037/a0021338 21319908

[43] Goodman MS, Madni LA, Semple RJ. Measuring mindfulness in youth: review of current assessments, challenges, and future directions. Mindfulness. 2017;8(6):1409–20. doi: 10.1007/s12671-017-0719-9

[44] Taylor A, Wright K, Roberts RM, Proeve M, Turner J, Miller C. Prevention of internalizing difficulties in the middle years: protocol for a noninferiority randomized trial of Mindfulness-Based Cognitive Therapy for Children and Cognitive Behavioural Therapy. Early Interv Psychiatry. 2024;18(7):547–52. doi: 10.1111/eip.13501 38318942

[45] Bakhtiari M, Habibi Asgarabad M, Dehghani F, Alhosseini KA, Semple RJ. Effectiveness and satisfaction of mindfulness-based cognitive therapy for children on anxiety, depression, and internet addiction in adolescents: Study protocol for a randomized control trial. PLoS One. 2025;20(4):e0317824. doi: 10.1371/journal.pone.0317824 40203011

[46] Kholghi H, Habibi Asgarabad M, Abolmaali Alhosseini K, Dehghani F, Semple RJ. The effectiveness of mindfulness-based cognitive therapy for children on anxiety, attentional control, and emotion regulation in children: a study protocol. Front Pediatr. 2025;13:1459434. doi: 10.3389/fped.2025.1459434 40787012 PMC12331755

[47] Lee JH, Dunner DL. The effect of anxiety disorder comorbidity on treatment resistant bipolar disorders. Depress Anxiety. 2008;25(2):91–7. doi: 10.1002/da.20279 17311265

[48] Cotton S, Kraemer KM, Sears RW, Strawn JR, Wasson RS, McCune N, et al. Mindfulness-based cognitive therapy for children and adolescents with anxiety disorders at-risk for bipolar disorder: a psychoeducation waitlist controlled pilot trial. Early Interv Psychiatry. 2020;14(2):211–9. doi: 10.1111/eip.12848 31264800 PMC7307795

[49] Harkness S, Gregg P, Fernández-Salgado M. The rise in single-mother families and children’s cognitive development: evidence from three british birth cohorts. Child Dev. 2020;91(5):1762–85. doi: 10.1111/cdev.13342 31745985 PMC9328442

[50] Semple RJ, Lee J. Chapter 8 - Mindfulness-Based Cognitive Therapy for Children. In: Baer RA, editor. Mindfulness-Based Treatment Approaches (Second Edition). San Diego: Academic Press; 2014. pp. 161–88.

[51] Porter B, Oyanadel C, Betancourt I, Worrell FC, Peñate W. Effects of two online mindfulness-based interventions for early adolescents for attentional, emotional, and behavioral self-regulation. Pediatric Rep. 2024;16(2):254–70. 10.3390/pediatric16020022 PMC11036234 38651461

[52] Galante J, Friedrich C, Collaboration of Mindfulness Trials (CoMinT) 3, Dalgleish T, Jones PB, White IR, et al. Individual participant data systematic review and meta-analysis of randomised controlled trials assessing adult mindfulness-based programmes for mental health promotion in non-clinical settings. Nat Ment Health. 2023;1(7):462–76. doi: 10.1038/s44220-023-00081-5 37867573 PMC7615230

[53] Butterfield KM, Roberts KP. The role of executive function in children’s mindfulness experience. Mindfulness. 2021;13(2):398–408. doi: 10.1007/s12671-021-01802-6

[54] Podsakoff PM, MacKenzie SB, Lee J-Y, Podsakoff NP. Common method biases in behavioral research: a critical review of the literature and recommended remedies. J Appl Psychol. 2003;88(5):879–903. doi: 10.1037/0021-9010.88.5.879 14516251

[55] Higgins JP, Green S. Cochrane handbook for systematic reviews of interventions. England: Cochrane Collaboration and John Wiley & Sons Ltd; 2008.

[56] Kazdin AE. Addressing the treatment gap: a key challenge for extending evidence-based psychosocial interventions. Behav Res Ther. 2017;88:7–18. doi: 10.1016/j.brat.2016.06.004 28110678

