# Supplementary material for: Mindfulness-based cognitive therapy for children (MBCT-C) on internalizing, externalizing, and attention problems in children from single-parent families: A study protocol for a crossover randomized clinical trial
Source: PLoS One. 2026 Aug 6;21(8):e0354802. doi: 10.1371/journal.pone.0354802 (PMC13446653; doi:10.1371/journal.pone.0354802)
Supplement: Supplementary file 1 [file pone.0354802.s001.pdf]

## Protocol summary

| | |
|---|---|
| **Study aim** | The purpose is to examining the effectiveness of Mindfulness-Based Cognitive Therapy for Children on internalizing and externalizing symptoms and attention problems in single-parent children. |
| **Design** | The clinical trial will have a control group, a blind strain and randomized on 76 patients |
| **Settings and conduct** | The location of the study will be in Kashan and the research will be conducted on single-parent children aged 8 to 12 years |
| **Participants/Inclusion and exclusion criteria** | The participants of this study will include children aged 8 to 12 years old. inclusion criteria: age between 8 and 12 years, not suffering from chronic psychiatric disorders, being a single parent family and the interest of the participants to participate in the research will be based on the written consent of the parent. exclusion criteria: a) unwillingness of the child's parent to participate in the sessions, b) changing the status of the child to no parent or two parents, c) missing more than two intervention sessions, d) the child suffering from a disease Chronic medical conditions that affect the child ability to attend meetings. |
| **Intervention groups** | The first group receives cognitive therapy based on mindfulness - child's version, and the second group receives the framework of mindfulness, without the content of mindfulness. Both treatments will have the same time and the same number of sessions. Then the second stage evaluation will be done. After the evaluation in the second stage, the first group will not receive any type of treatment and the second group will receive cognitive therapy based on mindfulness - child's version. Then re-evaluations will be done based on the tool. Then, two three-month periods of follow-up will be done for each group (six months in total). |
| **Main outcome variables** | Internalizing symptoms; Externalizing and attention problems |

## General information

| | |
|---|---|
| **Reason for update** | |
| **Acronym** | |
| **IRCT registration information** | IRCT registration number: **IRCT20240729062574N1**<br>Registration date: **2024-09-04, 1403/06/14**<br>Registration timing: **prospective**<br>Last update: **2024-09-07, 1403/06/17**<br>Update count: **1** |
| **Registration date** | 2024-09-04, 1403/06/14 |

| | |
|---|---|
| **Registrant information** | **Name**<br>Sara Jamali<br>**Name of organization / entity**<br>**Country**<br>Iran (Islamic Republic of)<br>**Phone**<br>+98 23 3362 3300<br>**Email address**<br>saraj4111@gmail.com |

| | |
|---|---|
| **Recruitment status** | **Recruitment complete** |
| **Funding source** | |

| | |
|---|---|
| **Expected recruitment start date** | 2024-09-22, 1403/07/01 |
| **Expected recruitment end date** | 2024-10-22, 1403/08/01 |
| **Actual recruitment start date** | *empty* |
| **Actual recruitment end date** | *empty* |
| **Trial completion date** | *empty* |

## General information

| | |
|---|---|
| **Reason for update** | |
| **Acronym** | |
| **IRCT registration information** | IRCT registration number: **IRCT20240729062574N1**<br>Registration date: **2024-09-04, 1403/06/14**<br>Registration timing: **prospective**<br>Last update: **2024-09-07, 1403/06/17**<br>Update count: **1** |
| **Registration date** | 2024-09-04, 1403/06/14 |

| | |
|---|---|
| **Registrant information** | **Name**<br>Sara Jamali<br>**Name of organization / entity**<br>**Country**<br>Iran (Islamic Republic of)<br>**Phone**<br>+98 23 3362 3300<br>**Email address**<br>saraj4111@gmail.com |

| | |
|---|---|
| **Recruitment status** | **Recruitment complete** |
| **Funding source** | |

| | |
|---|---|
| **Expected recruitment start date** | 2024-09-22, 1403/07/01 |
| **Expected recruitment end date** | 2024-10-22, 1403/08/01 |
| **Actual recruitment start date** | *empty* |
| **Actual recruitment end date** | *empty* |
| **Trial completion date** | *empty* |

| | |
|---|---|
| **Scientific title** | The effectiveness of Mindfulness-Based Cognitive Therapy for Children (MBCT-C) on internalizing and externalizing symptoms and attention problems in single-parent children |

| | |
|---|---|
| **Public title** | The effectiveness of Mindfulness-Based Cognitive Therapy for Children (MBCT-C) on internalizing and externalizing symptoms and attention problems in single-parent children |
| **Purpose** | Treatment |
| **Inclusion/Exclusion criteria** | **Inclusion criteria:**<br>Age between 8 and 12 years Not suffering from chronic psychiatric disorders (schizophrenia spectrum, bipolar spectrum and neurodevelopment disorders based on DSM.5) Being a single parent family Interest of the participants to participate in the research will be based on the written consent of the parent.<br>**Exclusion criteria:**<br>Unwillingness of the child's parent to participate in the sessions, Changing the status of the child to no parent or two parents, Missing more than two intervention sessions, The child suffering from a disease or chronic medical conditions that affect the child's ability to attend meetings. |
| **Age** | From **8 years** old to **12 years** old |
| **Gender** | Both |

| | |
|---|---|
| **Phase** | N/A |
| **Groups that have been masked** | • Participant |
| **Sample size** | Target sample size: **80** |
| **Randomization (investigator's opinion)** | Randomized |
| **Randomization description** | A list will be made of the participants and they will be matched in pairs based on age, and they will be labeled A and B and will be divided into two groups using a table of random numbers. 80 children will age and gender-matched and then randomly will assign to MBCT group and sham mindfulness group. The MBCT-C group participants will divide into 4 small group. |
| **Blinding (investigator's opinion)** | Single blinded |
| **Blinding description** | Considering that the researcher wants to implement the treatment, they will know how to implement the treatment and the participants are unaware of the information and the treatment method of the other group. |
| **Placebo** | Used |
| **Assignment** | Crossover |
| **Other design features** | |

## Secondary Ids

*empty*

## Ethics committees

1

| | |
|---|---|
| **Ethics committee** | **Name of ethics committee**<br>Ethics committee of Semnan University of Medical Science<br>**Street address**<br>Headquarter of Semnan University of Medical Sciences and Health Services, Bassij Blvd, Semnan, Iran.<br>**City**<br>Semnan<br>**Province**<br>Semnan<br>**Postal code**<br>35147-99442 |
| **Approval date** | 2024-07-10, 1403/04/20 |
| **Ethics committee reference number** | IR.SEMUMS.REC.1403.046 |
