# Supplementary material for: Mindfulness-based cognitive therapy for children (MBCT-C) on internalizing, externalizing, and attention problems in children from single-parent families: A study protocol for a crossover randomized clinical trial
Source: PLoS One. 2026 Aug 6;21(8):e0354802. doi: 10.1371/journal.pone.0354802 (PMC13446653; doi:10.1371/journal.pone.0354802)
Supplement: Supplementary file 2 [file pone.0354802.s002.pdf]

چکیده پروتکل

**هدف ازمطالعه**	هدف از پژوهش حاضر، بررسی اثربخشی شناخت درمانی مبتنی بر ذهن آگاهی کودکان بر علائم درونی سازی؛ برونی سازی و مشکلات توجه در کودکان تک سرپرست، است.
**طراحی**	کارآزمایی بالینی دارای گروه کنترل، یک سویه کور و تصادفی سازی شده بر روی 76 بیمارخواهد بود
**نحوه و محل انجام مطالعه**	محل انجام مطالعه در شهرستان کاشان و انجام پژوهش بر روی کودکان تک سرپرست 8 تا 12 ساله خواهد بود
**شرکت کنندگان/شرایط ورود و عدم ورود**	شرایط ورود: سن بین 8 تا 12 سال، عدم ابتلا به اختلالات روانپزشکی مزمن (طیف اسکیزوفرنیا، طیف دو قطبی، و اختلالات عصبی-رشدی بر مبنای DSM.5) ، تک سرپرست بودن کودک و علاقه مندی شرکت کنندگان برای مشارکت در پژوهش بر اساس رضایت نامه کتبی والد<br>شرایط عدم ورود: الف)عدم تمایل سرپرست کودک به مشارکت کودک در ادامه جلسات، ب) تغییر وضعیت کودک به بدون سرپرست/دو سرپرست ، ج) عدم شرکت در بیش از دو جلسه از مداخله، د)ابتلای کودک به یک بیماری طبی مزمن که شرایط کودک برای حضور در جلسات را تحت تاثیر قرار دهد
**گروههای مداخله**	گروه اول شناخت درمانی مبتنی بر ذهن آگاهی -نسخه کودک و گروه دوم چارچوب ذهن اگاهی، فاقد محتوای ذهن آگاهی را دریافت می کنند. هر دو درمان دارای زمان یکسان و تعداد جلسات یکسان خواهند بود. سپس ارزیابی مرحله دوم انجام خواهد شد. پس از ارزیابی در مرحله دوم، گروه اول هیچ نوع درمانی دریافت نخواهد کرد و گروه دوم شناخت درمانی مبتنی بر ذهن آگاهی -نسخه کودک را دریافت خواهند کرد. سپس مجددا ارزیابی ها براساس ابزار انجام خواهد شد. سپس دو بازه سه ماهه پیگیری برای هر گروه (مجموعا شش ماه) انجام خواهد شد.
**متغیرهای پیامد اصلی**	علائم درونی سازی؛ برونی سازی و مشکلات توجه

اطلاعات عمومی

**علت بروز رسانی**	
**نام اختصاری**	
**اطلاعات ثبت در مرکز**	شماره ثبت کارآزمایی در مرکز: **IRCT20240729062574N1**<br>تاریخ تایید ثبت در مرکز: **2024-09-04, ۱۴۰۳/۰۶/۱۴**<br>زمان‌بندی ثبت: **prospective**<br>آخرین بروز رسانی: **2024-09-07, ۱۴۰۳/۰۶/۱۷**<br>تعداد بروز رسانی‌ها: **1**
**تاریخ تایید ثبت در مرکز**	2024-09-04, ۱۴۰۳/۰۶/۱۴

**اطلاعات تماس ثبت کننده**	**نام**<br>سارا جمالی<br>**نام سازمان / نهاد**<br>**کشور**<br>جمهوری اسلامی ایران<br>**تلفن**<br>+98 23 3362 3300<br>**آدرس ایمیل**<br>saraj4111@gmail.com

**وضعیت بیمار گیری**	**بیمار گیری تمام شده**
**منبع مالی**	

**تاریخ شروع بیمار گیری مورد انتظار**	2024-09-22, ۱۴۰۳/۰۷/۰۱
**تاریخ پایان بیمار گیری مورد انتظار**	2024-10-22, ۱۴۰۳/۰۸/۰۱
**تاریخ شروع بیمارگیری تحقق یافته**	خالی
**تاریخ پایان بیمارگیری تحقق یافته**	خالی
**تاریخ خاتمه کارآزمایی**	خالی

**عنوان علمی کارآزمایی**	اثربخشی شناخت درمانی مبتنی بر ذهن آگاهی کودکان بر علائم درونی سازی؛ برونی سازی و مشکلات توجه در کودکان تک سرپرست

**عنوان عمومی کارآزمایی**	اثربخشی شناخت درمانی مبتنی بر ذهن آگاهی کودکان بر علائم درونی سازی؛ برونی سازی و مشکلات توجه در کودکان تک سرپرست
**هدف اصلی مطالعه**	درمانی
**شرایط عمده ورود و عدم ورود به مطالعه**	**شرایط عمده ورود به مطالعه قبل از تصادفی سازی:**<br>سن بین 8 تا 12 سال عدم ابتلا به اختلالات روانپزشکی مزمن (طیف اسکیزوفرنیا، طیف دو قطبی، و اختلالات عصبی-رشدی بر مبنای DSM.5)

**وضعیت بیمار گیری**	**بیمار گیری تمام شده**
**منبع مالی**	

**تاریخ شروع بیمار گیری مورد انتظار**	2024-09-22, ۱۴۰۳/۰۷/۰۱
**تاریخ پایان بیمار گیری مورد انتظار**	2024-10-22, ۱۴۰۳/۰۸/۰۱
**تاریخ شروع بیمارگیری تحقق یافته**	خالی
**تاریخ پایان بیمارگیری تحقق یافته**	خالی
**تاریخ خاتمه کارآزمایی**	خالی

**عنوان علمی کارآزمایی**	اثربخشی شناخت درمانی مبتنی بر ذهن آگاهی کودکان بر علائم درونی سازی؛ برونی سازی و مشکلات توجه در کودکان تک سرپرست

**عنوان عمومی کارآزمایی**	اثربخشی شناخت درمانی مبتنی بر ذهن آگاهی کودکان بر علائم درونی سازی؛ برونی سازی و مشکلات توجه در کودکان تک سرپرست
**هدف اصلی مطالعه**	درمانی
**شرایط عمده ورود و عدم ورود به مطالعه**	**شرایط عمده ورود به مطالعه قبل از تصادفی سازی:**<br>سن بین 8 تا 12 سال عدم ابتلا به اختلالات روانپزشکی مزمن (طیف اسکیزوفرنیا، طیف دو قطبی، و اختلالات عصبی-رشدی بر مبنای DSM.5) تک سرپرست بودن کودک علاقه مندی شرکت کنندگان بر اساس رضایت کتبی والد<br>**شرایط عمده عدم ورود به مطالعه قبل از تصادفی سازی:**<br>عدم تمایل سرپرست کودک به مشارکت کودک در ادامه جلسات تغییر وضعیت کودک به بدون سرپرست/دو سرپرست عدم شرکت در بیش از دو جلسه از مداخله ابتلای کودک به یک بیماری طبی مزمن
**سن**	از سن **8 ساله** تا سن **12 ساله**
**جنسیت**	هر دو

**فاز مطالعه**	مصداق ندارد
**گروه‌های کور شده در مطالعه**	• شرکت کننده
**حجم نمونه کل**	حجم نمونه پیش‌بینی شده: **80**
**تصادفی سازی (نظر محقق)**	اختصاص تصادفی به گروه‌های مداخله و کنترل
**توصیف نحوه تصادفی سازی**	از افراد شرکت کننده لیستی تهیه شد و بر اساس سن و به صورت دوتایی همتاسازی صورت گرفت و برچسب الف و ب می گیرند و به وسیله جدول اعداد تصادفی به دو گروه تقسیم می شوند. 80 کودک از نظر سنی و جنسیت همسان شده و سپس به طور تصادفی در گروه MBCT و گروه ذهن آگاهی ساختگی قرار خواهند گرفت. شرکت کنندگان گروه MBCT-C به 4 گروه کوچک تقسیم می شوند. سپس درمان بر روی آنها توسط درمانگر مجرب اجرا خواهد شد
**کور سازی (به نظر محقق)**	یک سویه کور
**توصیف نحوه کور سازی**	با توجه به اینکه پژوهشگر میخواهد درمان را اجرا کند از نحوه اجرای درمان اطلاع خواهند داشت و شرکت کنندگان از اطلاعات و نحوه درمان گروه دیگر بی اطلاع هستند
**دارو نما**	دارد
**اختصاص به گروه‌های مطالعه**	متقاطع
**سایر مشخصات طراحی مطالعه**	

تاییدیه کمیته‌های اخلاق

1	
**کمیته اخلاق**	**نام کمیته اخلاق**<br>کمیته اخلاق دانشگاه علوم پزشکی سمنان<br>**آدرس خیابان**<br>سمنان بلوار بسیج ستاد دانشگاه علوم پزشکی و خدمات بهداشتی درمانی استان سمنان<br>**شهر**<br>سمنان<br>**استان**<br>سمنان<br>**کد پستی**<br>35147-99442
**تاریخ تایید**	2024-07-10, ۱۴۰۳/۰۴/۲۰
**کد کمیته اخلاق**	IR.SEMUMS.REC.1403.046

بیماری‌های (موضوعات) مورد مطالعه

1	
**شرح**	علائم درونی سازی
**کد ICD-10**	
**توصیف کد ICD-10**	

2	
**شرح**	علائم برونی سازی
**کد ICD-10**	
**توصیف کد ICD-10**	

3	
**شرح**	مشکلات توجه
**کد ICD-10**	
**توصیف کد ICD-10**	

متغیر پیامد اولیه

1	
**شرح متغیر پیامد**	علائم درون سازی
**مقاطع زمانی اندازه‌گیری**	قبل از اجرای مداخله، هر دو گروه مورد ارزیابی اولیه (خط پایه قرار می گیرند). سپس گروه اول شناخت درمانی مبتنی بر ذهن آگاهی -نسخه کودک و گروه دوم چارچوب ذهن اگاهی، فاقد محتوای ذهن آگاهی را دریافت می کنند. هر دو درمان دارای زمان یکسان و تعداد جلسات یکسان خواهند بود. سپس ارزیابی مرحله دوم انجام خواهد شد. پس از ارزیابی در مرحله دوم، گروه اول هیچ نوع درمانی دریافت نخواهد کرد و گروه دوم شناخت درمانی مبتنی بر ذهن آگاهی -نسخه کودک را دریافت خواهند کرد. سپس مجددا ارزیابی ها براساس ابزار انجام خواهد شد. سپس دو بازه سه ماهه پیگیری برای هر گروه (مجموعا شش ماه) انجام خواهد شد.
**نحوه اندازه‌گیری متغیر**	نحوه اندازه گیری متغیرها، استفاده از پرسشنامه مقیاس مشکلات رفتاري کودک و نوجوان، خواهد بود

2	
**شرح متغیر پیامد**	علائم برون سازی
**مقاطع زمانی اندازه‌گیری**	قبل از اجرای مداخله، هر دو گروه مورد ارزیابی اولیه (خط پایه قرار می گیرند). سپس گروه اول شناخت درمانی مبتنی بر ذهن آگاهی -نسخه کودک و گروه دوم چارچوب ذهن اگاهی، فاقد محتوای ذهن آگاهی را دریافت می کنند. هر دو درمان دارای زمان یکسان و تعداد جلسات یکسان خواهند بود. سپس ارزیابی مرحله دوم انجام خواهد شد. پس از ارزیابی در مرحله دوم، گروه اول هیچ نوع درمانی دریافت نخواهد کرد و گروه دوم شناخت درمانی مبتنی بر ذهن آگاهی -نسخه کودک را دریافت خواهند کرد. سپس مجددا ارزیابی ها براساس ابزار انجام خواهد شد. سپس دو بازه سه ماهه پیگیری برای هر گروه (مجموعا شش ماه) انجام خواهد شد.
**نحوه اندازه‌گیری متغیر**	نحوه اندازه گیری متغیر، استفاده از مقیاس مشکلات رفتاري کودک و نوجوان خواهد بود

متغیر پیامد اولیه

1	
شرح متغیر پیامد	علائم درون سازی
مقاطع زمانی اندازه‌گیری	قبل از اجرای مداخله، هر دو گروه مورد ارزیابی اولیه (خط پایه قرار می گیرند). سپس گروه اول شناخت درمانی مبتنی بر ذهن آگاهی -نسخه کودک و گروه دوم چارچوب ذهن اگاهی، فاقد محتوای ذهن آگاهی را دریافت می کنند. هر دو درمان دارای زمان یکسان و تعداد جلسات یکسان خواهند بود. سپس ارزیابی مرحله دوم انجام خواهد شد. پس از ارزیابی در مرحله دوم، گروه اول هیچ نوع درمانی دریافت نخواهد کرد و گروه دوم شناخت درمانی مبتنی بر ذهن آگاهی -نسخه کودک را دریافت خواهند کرد. سپس مجددا ارزیابی ها براساس ابزار انجام خواهد شد. سپس دو بازه سه ماهه پیگیری برای هر گروه (مجموعا شش ماه) انجام خواهد شد.
نحوه اندازه‌گیری متغیر	نحوه اندازه گیری متغیرها، استفاده از پرسشنامه مقیاس مشکلات رفتاري کودک و نوجوان، خواهد بود

2	
شرح متغیر پیامد	علائم برون سازی
مقاطع زمانی اندازه‌گیری	قبل از اجرای مداخله، هر دو گروه مورد ارزیابی اولیه (خط پایه قرار می گیرند). سپس گروه اول شناخت درمانی مبتنی بر ذهن آگاهی -نسخه کودک و گروه دوم چارچوب ذهن اگاهی، فاقد محتوای ذهن آگاهی را دریافت می کنند. هر دو درمان دارای زمان یکسان و تعداد جلسات یکسان خواهند بود. سپس ارزیابی مرحله دوم انجام خواهد شد. پس از ارزیابی در مرحله دوم، گروه اول هیچ نوع درمانی دریافت نخواهد کرد و گروه دوم شناخت درمانی مبتنی بر ذهن آگاهی -نسخه کودک را دریافت خواهند کرد. سپس مجددا ارزیابی ها براساس ابزار انجام خواهد شد. سپس دو بازه سه ماهه پیگیری برای هر گروه (مجموعا شش ماه) انجام خواهد شد.
نحوه اندازه‌گیری متغیر	نحوه اندازه گیری متغیر، استفاده از مقیاس مشکلات رفتاري کودک و نوجوان خواهد بود

3	
شرح متغیر پیامد	مشکلات توجه
مقاطع زمانی اندازه‌گیری	قبل از اجرای مداخله، هر دو گروه مورد ارزیابی اولیه (خط پایه قرار می گیرند). سپس گروه اول شناخت درمانی مبتنی بر ذهن آگاهی -نسخه کودک و گروه دوم چارچوب ذهن اگاهی، فاقد محتوای ذهن آگاهی را دریافت می کنند. هر دو درمان دارای زمان یکسان و تعداد جلسات یکسان خواهند بود. سپس ارزیابی مرحله دوم انجام خواهد شد. پس از ارزیابی در مرحله دوم، گروه اول هیچ نوع درمانی دریافت نخواهد کرد و گروه دوم شناخت درمانی مبتنی بر ذهن آگاهی -نسخه کودک را دریافت خواهند کرد. سپس مجددا ارزیابی ها براساس ابزار انجام خواهد شد. سپس دو بازه سه ماهه پیگیری برای هر گروه (مجموعا شش ماه) انجام خواهد شد.
نحوه اندازه‌گیری متغیر	نحوه اندازه گیری متغیر، استفاده از چک لیست علائم کودکان - گزارش کودک (بخش توجه) خواهد بود

متغیر پیامد ثانویه

خالی

گروه‌های مداخله

1	
شرح مداخله	گروه مداخله: درمان مبتنی بر ذهن آگاهی کودکان شامل 12 جلسه مداخله 45 دقیقه ای خواهد بود. محتوای جلسات طبق پروتوکل شامل آموزش تکنیک های ذهن آگاهی مانند نفس کشیدن ذهن آگاهانه، چشیدن ذهن آگاهانه، گوش دادن ذهن آگاهانه، لمس کردن ذهن آگاهانه و مشاهده کردن و تجربه ذهن آگاهانه خواهد بود.
طبقه بندی	درمانی - غیره

2	
شرح مداخله	گروه کنترل: برنامه ذهن آگاهی ساختگی که برای شرکت کنندگان در شرایط ساختگی در دسترس است به عنوان "مراقبه" و تمرینات به عنوان "مدیتیشن" توصیف می شود. به‌جای تکنیک‌های مبتنی بر ذهن‌آگاهی که در محیط درمان مورد استفاده قرار می‌گرفت، برنامه ذهن‌آگاهی ساختگی به طور متناوب بین چهار تکنیک مختلف که به طور خلاصه تنفس شرکت‌کنندگان را هدایت می‌کرد، بدون ارائه دستورالعمل‌هایی برای مشاهده آشکار و بدون قضاوت احساسات و افکار، جایگزین شد.
طبقه بندی	دارو نما

2	
**شرح مداخله**	گروه کنترل: برنامه ذهن آگاهی ساختگی که برای شرکت کنندگان در شرایط ساختگی در دسترس است به عنوان "مراقبه" و تمرینات به عنوان "مدیتیشن" توصیف می شود. به‌جای تکنیک‌های مبتنی بر ذهن‌آگاهی که در محیط درمان مورد استفاده قرار می‌گرفت، برنامه ذهن‌آگاهی ساختگی به طور متناوب بین چهار تکنیک مختلف که به طور خلاصه تنفس شرکت‌کنندگان را هدایت می‌کرد، بدون ارائه دستورالعمل‌هایی برای مشاهده آشکار و بدون قضاوت احساسات و افکار، جایگزین شد.
**طبقه بندی**	دارو نما

مراکز بیمار گیری

1	
**مرکز بیمار گیری**	**نام مرکز بیمار گیری**<br>موسسه خیریه بنیاد کودک<br>**نام کامل فرد مسوول**<br>سارا جمالی<br>**آدرس خیابان**<br>کاشان، خیابان شهید رجایی<br>**شهر**<br>کاشان<br>**استان**<br>اصفهان<br>**کد پستی**<br>8713753963<br>**تلفن**<br>+98 31 5546 9898<br>**ایمیل**<br>sarajamalii1400@gmail.com

حمایت کنندگان / منابع مالی

1	
**حمایت کننده مالی**	**نام سازمان / نهاد**<br>معاونت پژوهشی دانشگاه سمنان<br>**نام کامل فرد مسوول**<br>دکتر محمود نجفی<br>**آدرس خیابان**<br>سمنان، مهدیشهر، دربند، دانشکده روان شناسي وعلوم تربيتي مهديشهر، دانشگاه سمنان<br>**شهر**<br>سمنان<br>**استان**<br>سمنان<br>**کد پستی**<br>3564111556<br>**تلفن**<br>+98 23 3153 3018<br>**فکس**<br>**ایمیل**<br>m_najafi@semnan.ac.ir
ردیف بودجه	
کد بودجه	
**آیا منبع مالی همان سازمان یا نهاد حمایت کننده مالی است؟**	بلی
**عنوان منبع مالی**	معاونت پژوهشی دانشگاه سمنان
**درصد تامین مالی مطالعه توسط این منبع**	100
**بخش عمومی یا خصوصی**	عمومی
**مبدا اعتبار از داخل یا خارج کشور**	داخلی
**طبقه بندی منابع اعتبار خارجی**	*خالی*
**کشور مبدا**	
**طبقه بندی موسسه تامین کننده اعتبار**	دانشگاهی

فرد مسوول پاسخگویی عمومی کارآزمایی

اطلاعات تماس

نام سازمان / نهاد
دانشگاه علوم پزشکی سمنان

نام کامل فرد مسوول
سارا جمالی

موقعیت شغلی
دانشجوی دکتری روانشناسی

آخرین مدرک تحصیلی
فوق لیسانس

سایر حوزه‌های کاری/تخصص‌ها
روانشناسی

آدرس خیابان
سمنان، دانشگاه سمنان، دانشکده روانشناسی و علوم تربیتی، گروه روانشناسی بالینی

شهر
سمنان

استان
سمنان

کد پستی
8719667996

تلفن
+98 23 3362 3300

فکس
+98 23 3362 5554

ایمیل
saraj4111@gmail.com

فرد مسوول پاسخگویی علمی مطالعه

اطلاعات تماس

نام سازمان / نهاد
دانشگاه سمنان

نام کامل فرد مسوول
اسحق رحیمیان بوگر

موقعیت شغلی
دانشیار گروه آموزشی روانشناسی

آخرین مدرک تحصیلی
Ph.D.

سایر حوزه‌های کاری/تخصص‌ها
روانشناسی

آدرس خیابان
سمنان، دانشگاه سمنان، دانشکده روانشناسی و علوم تربیتی، گروه روانشناسی بالینی

شهر
سمنان

استان
سمنان

کد پستی
8719667996

تلفن
+98 23 3362 3300

فکس
+98 23 3362 5554

ایمیل
i_rahimian@semnan.ac.ir

آدرس صفحه وب
https://www.scopus.com/authid/detail.uri?authorId=54893135700

فرد مسوول به‌روز رسانی اطلاعات

اطلاعات تماس

نام سازمان / نهاد
انستیتو پاستور ایران

نام کامل فرد مسوول
مجتبی حبیبی عسگرآباد

موقعیت شغلی
استادیار

آخرین مدرک تحصیلی
Ph.D.

سایر حوزه‌های کاری/تخصص‌ها
روانشناسی

آدرس خیابان
تهران، بلوار ستارخان، خیابان نیایش، خیابان منصوری
