# Supplementary material for: Mindfulness-based cognitive therapy for children (MBCT-C) on internalizing, externalizing, and attention problems in children from single-parent families: A study protocol for a crossover randomized clinical trial
Source: PLoS One. 2026 Aug 6;21(8):e0354802. doi: 10.1371/journal.pone.0354802 (PMC13446653; doi:10.1371/journal.pone.0354802)
Supplement: Supplementary file 3 [file pone.0354802.s004.docx]

Inclusivity in global research

PLOS’ policy on inclusivity in global research aims to improve transparency in the reporting of research performed outside of researchers’ own country or community and ensures that PLOS publications reporting global research adhere to high standards for research ethics and authorship. Authors of relevant research articles may be asked to complete the questionnaire below, which outlines ethical, cultural, and scientific considerations specific to inclusivity in global research. This questionnaire may be requested when researchers have travelled to a different country to conduct research, if research uses samples collected in another country, research with Indigenous populations or their lands, or if research is on cultural artefacts. Researchers travelling to another country solely to use laboratory equipment will not normally be required to complete the questionnaire. However, the questionnaire can be requested at the journal’s discretion for any submission – if you have been requested to complete this questionnaire by the PLOS journal you submitted to, please do so.

Please complete the questionnaire below and include this as a Supporting Information file with your manuscript. Note that if your paper is accepted for publication, this checklist will be published with your article in the supporting information files. Please ensure that you reference the checklist in the main body of your manuscript. We suggest adding a subsection ‘Inclusivity in global research’ to your Methods section and adding the following sentence: “Additional information regarding the ethical, cultural, and scientific considerations specific to inclusivity in global research is included in the Supporting Information (SX Checklist)”

The questions have been designed to be applicable to a wide range of study types, and there are subsections for both human subjects research and non-human subjects research. If any of the questions are not relevant to your research please mark them as “N/A” as appropriate.

**Ethical considerations, permits and authorship**

*This section is applicable to all research types.*

Provide details as to who granted permissions and/or consent for the study to take place in the Methods section of your manuscript. This should include the names of **all** ethics boards, governmental organizations, community leaders or other bodies that provided approval for the study. If individuals provided approval refer to these people by their role or title but do not list their name(s).

Reported on page number: p 17-18

If there were any deviations from the study protocol after approval was obtained please provide details of these changes in the Methods section of your manuscript.
Did this study involve local collaborators that are residents of the country where the research was conducted or members of the community studied? If you do not have any authors from said communities, please provide an explanation for this below.

Reported on page number: p 19

Yes, all authors except Dr. Randye J. Semple (USA citizen) and Dr. Mojtaba Habibi Asgharabad (Iranian citizen living in Norway) currently reside in Iran.

Everyone listed as an author should meet PLOS’ criteria for authorship and all individuals who meet these criteria should be included in the author byline, rather than the acknowledgements. For further information please see the journal’s Authorship Policy.

**Human subjects research (e.g. health research, medical research, cross-cultural psychology)**

Did you obtain written informed consent from a representative of the local community or region before the research took place? How did you establish who speaks for the community? Details of written informed consent obtained from study participants should be reported separately in the Methods section of your manuscript.

The informed consent form and assent will include comprehensive information about the study's purpose, the methods and types of interventions to be employed, and the criteria for participant inclusion and exclusion. We will clearly outline the potential risks and benefits associated with participation, as well as the measures we will implement to protect participants' privacy. Additionally, we will emphasize the right to withdraw from the study at any time and provide contact information for any questions or concerns. Importantly, we will ensure that parental or guardian consent is obtained, allowing parents to make an informed decision regarding their children's participation in our study

How did members of the local community provide input on the aims of the research investigation, its methodology, and its anticipated outcome(s)?

The consultations with school leaders were essential to shaping our research design, ensuring that our approach was both suitable for the educational setting and responsive to the unique needs of the students. Through discussions with school representatives about our objectives and methodologies, we aimed to ensure our study was relevant and ethically sound, honoring the community's values and expectations. These collaborative discussions were crucial in improving our tactics to decrease dropout rates and enhance participant engagement throughout the research.

When engaging with the local community, how did you ensure that the informed consent documents and other materials could be understood by local stakeholders?

We are a research team deeply rooted in the society we are studying, giving us valuable insights into its social and cultural dynamics. To ensure that our informed consent documents and other materials are clear and understandable to local stakeholders, we have taken several important steps:

First, we focused on language and terms. We made a conscious effort to use simple language and avoid any jargon or technical jargon that could confuse people. In addition, we translated all documents into the local language to ensure that everyone in the community can access and understand the information.

In the next step, we prioritized community participation. We worked closely with school administrators and parents during the development of our study. Their feedback was invaluable and helped us shape the content to better align with the community's values and context.

We also created a feedback mechanism. By holding focus groups and talking to community members, we were able to collect their opinions on the documents. This process helped us identify any unclear areas and make necessary adjustments based on their input.

Finally, we organized training sessions where we guided participants through the consent process and answered their questions. These face-to-face interactions were critical to alleviating any concerns and ensuring that stakeholders felt comfortable with the information we were providing.

Will the findings of the research be made available in an understandable format to stakeholders in the community where the study was conducted (e.g. via a presentation, summary report, copies of publications, etc.)? Please provide details of how this will be achieved.

We will ensure that our research findings are accessible to community stakeholders in a way that is easy to understand. Our goal is to make the results meaningful to everyone involved, including those who participated in the study and the wider community. Here's how we plan to do it:

First, we create a summary report that distills the key findings into a concise format. This report will be written in simple language without technical terms so that all members of the community can easily understand the information.

Next, we will host community presentations where we can share our findings in an engaging and interactive way. These sessions provide an opportunity for stakeholders to ask questions and discuss directly with our research team.

In addition, we will use visual aids such as charts, infographics and slides during our presentation. These visual images help clarify the findings and make the information more relevant and easier to understand.

**Non-human subjects research using specimens/ animals collected as part of the study, or those housed in archival collections. Examples include archaeology, paleontology, botany and zoology.**

Did the permission you obtained from a local authority to perform the study include an agreement on access to outputs and benefit sharing? This may include procedures to enable fair distribution of the benefits and resources arising from the research performed. Please include any details of Prior Informed Consent and Benefit Sharing Agreements obtained. These may be required by field-specific regulations, for example the Convention on Biological Diversity (CBD) and the associated Nagoya Protocol.

Not applicable

If the material used in your study was imported, please A) provide the year it was imported and B) indicate whether permits were obtained to import/export the materials used, C) provide details of any permits obtained. If this information is not available, please indicate this.

Not applicable

If you used archival specimens, please state how the material used in your study was acquired by the institute it is held in and provide details of any permits obtained for the original excavations/ sample collection. If this information is not available, please indicate this.

Not applicable

How was the potential cultural significance of the materials collected in your study to local communities considered in your research design? Were Indigenous peoples and/or local researchers and institutions involved with archaeological excavations / collection of specimens? If so, please provide a description of their involvement.

Not applicable

If your manuscript includes photographs of human remains please indicate whether authors obtained permission from descendants or affiliated cultural communities to do so.

Not applicable
